# The Therapeutic Effects and Uses of Mercury, Etc.

**Published:** 1873-08

**Authors:** Wm. H. Doughty

**Affiliations:** Professor of Materia Medica and Therapeutics in the Medical College of Georgia


### THE THERAPEUTIC EFFECTS AND USES OF MERCURY,

### *AS INFLUENCED BY THE "REPORT OF THE EDINBURG COMMITTEE ON THE ACTION OF MERCURY, PODOPHYLLIN, AND TARAXACUM, ON THE BILIARY SECRETION."*

BY WM. H. DOUGHTY, M.D.,

*Professor of Materia Medica and Therapeutics in the Medical College of Georgia.*

[From the Transactions of the Medical Association of Georgia, 1873.]

Perhaps the most attractive feature in medicine, as at present taught and practiced, except its growing certainty, is its conservatism. Not only in surgery, but in therapeutics, it is so plainly seen that the injunction of Chomel to "treat the patient rather than the disease," may be regarded as in full force. The renewal of life, as contemplated in the cure of disease, is better accomplished by the conservation of the forces of the system by all proper means or measures, than by any system of medication bordering upon the exhaustive or extremely active. Under this ruling, venesection, mercury, and the *inevitable* purgative have been assigned a limited sphere of usefulness, while restoratives, (food and medicines), promoters of nutrition at large, eliminatives, and hygenic measures, have grown in favor with our increasing knowledge of disease.

For one, then, to resume the defense of mercury against what may be termed scientific encroachment (in contradistinction to empirical observation) is, perhaps, to incur a liability to the charge of retarding the progress of medicine. The time is not far distant when mercury (there was a power in the very name) was the talisman of the therapeutic art—the touchstone, by which all systems of practice were tested. The bold used, defended, and praised it, while the weak and thoughtless illustrated its claims and virtues by its universal administration. Now, however, the pendulum of medical opinion has swung to the opposite extreme: whereas, once it was mercury for everything, now it is almost mercury for nothing — indeed, even those effects and uses that can, from necessity, only be seen in its therapeutical employment meet with denial. It has manifestly lost its charm, and, like the barren fig-tree, is about to have the axe laid at its root, by the total denial of its *hepatic* virtues.

Several circumstances worthy of mention have contributed to this revolution in opinion. We recount them in the order of their importance: first, an improved pathology of disease, particularly that of fevers and inflamation; second, a better understanding of the properties of other and safer remedies, as quinine, and eliminatives generally; and, lastly, the abuse of mercury. What a fearful picture of horror does the contemplation of the past abuse or misuse of mercury unfold before the imagination! The spectres of the dead and diseased and degenerate, all victims to science, rise in an innumerable throng to convict the profession of the terrible responsibility of their acts. Well may we pause on the threshold of its defense. We would gladly cover the unpleasant spectacle with the veil of silence, but for the fear that something said in the present article might quicken its indiscriminate use by some who still give undeserved prominence to it as a therapeutical agent.

But there may be (as there have been in the far past) errors in conservatism. While it may be more pleasant to the patient, so far the as drugs to be taken is concerned, we are not sure that there may not be quite as much danger in too little as in overmuch medication. A satirical author* of the 17th century, in the supreme contempt which his system of medicine—Animism or Psycho-Vitalis—inspired for medicinal agents, characterized the Materia Medica as a "*stable full of offal.*" In the same sense that Sir John Forbes denominated Homœopathy, "Nature in therapeutic power," we may say of this, the literal *expectant* system, in its very beginning, it is *fanaticism in conservatism.* We fear that we are on the verge of its reproduction, when, at this enlightened day, clinical observation is so readily set at naught by limited experiments on lower animals. The latter may lead as readily to the adoption of errors, as may the former to a false interpretation of the phenomena of disease in connection with the use and effects of remedies. In the effort to escape the one, let us not run madly into the adoption of the other, but with the single motive of truth impelling us, the profession is called upon to consider whether the report of the Edinburg Committe, — adverse to the cholagogue effects of mercury,—is to be accepted as satisfactory and conclusive.

It may be proper to state that, after two years experimentation upon lower animals (dogs), this committee reported the results of their investigations through the chairman, Dr. John

* Stahl, of the University of Halle.

Hughes Bennett. They were well satisfied with their labors, having declared them to be "quite exhaustive, and to *leave nothing to be desired.*" The mode of investigation was that long employed by the physiologist in the study of the functions of the liver in its relations to disgestion, namely, by the establishment of biliary fistulæ. It is but fair to state, that the committee, in the conduct of these experiments, seem to have been animated by a desire to reach reliable conclusions, by eliminating as far as possible all sources of error, and by anticipating objections that might be urged by those who hesitated to accept them. In this sense, *as far as the question could be determined by such experiments*, it may be said, that they were a crucial test, submitting to physical measurement, and chemical and microscopic examination, the whole amount of bile, biliary salts, etc., secreted in twenty-four hours, before, during and after the administration of the articles experimented with. We see one conculsion —the only one that could be definitely reached by this mode of procedure—which is unquestionably established, namely, that mercury, when administered to healthy dogs, in sensible quantities, disturbs the action of the liver, in common with the whole system, which disturbance is manifested by a decrease of the bile secreted by that viscus. The *physiological action* of mercury then on the liver is a *diminution* of its secretion. They selected dogs because they were best suited for the observations to be made, having first satisfied themselves that they were similiary influenced to man by mercurials.

We will allow them to state their conclusions: "The foregoing observations," say they, "seem to us clearly to show that blue pill, calomel, and corrosive sublimate, when given to dogs in either small, gradually augmented, or in large doses, do not increase the biliary secretion; they do not *even influence* it so long as neither purgation nor impairment of health are produced, but they diminish it as soon as they produce either or both." Anticipating the objection that, possibly, it might have a different effect on man in health, after due and impartial consideration of the physiological conditions pertinent to both, as well as the difference in effect of medicinal agents on man and the lower animals, they "infer that it is in the highest degree probable that its action upon the hepatic secretion will also be the same." We think most persons will assent to the correctness of this probability. But, having felt their way cautiously to this point, beyond which vivisections could not carry them in order to give practical force and bearing to the conclusions thus experimentally reached, they remark further, as follows:

"But some may say that, although we have proved that mercury diminished the biliary secretion in dogs, and that in man its action will, in all probability, be the same, yet our experiments have been performed on animals in a state of health; and that, had they been made on dogs with diseases such as those in which mercury has been *supposed* to increase the hepatic secretion, it would, possibly, in the case of such dogs, have been increased. With such an hypothesis we need not seriously occupy ourselves until the objectors *prove* that, in any case whatever, mercury can increase the biliary secretion in man.

"We have been unable to discover any facts brought to light in this or any other age which prove that mercury stimulates the biliary secretion. So far as we can make out, the notion that it does so, originates in some vague statement made by Paracelsus, or the authors of his time, as to the good effects of mercury in what he has called 'icteritia.' But we repeat, not only do we not know how such a notion has arisen, but we are ignorant how to make direct observations on the subject in man. We have already stated that such observations are, in the present state of physiological chemistry, impossible. We do not deny the possibility of mercury being useful in some diseases of the liver. We simply say that the notion of its doing good by means of increasing the biliary secretion is untenable."

They conclude with the following observation:

"It is unnecessary to dwell upon the importance of the results at which the committee have taken so much pains to arrive. If the refutation of the wide-spread error be as important as the establishment of a new truth, the practical advantage of demonstrating that mercury is not a cholagogue can not be too highly estimated. Although in recent times the administration of mercurials for hepatic diseases has greatly diminished, their employment is still very general, and in India almost universal. Recent cases demonstrate that long-continued salivation and great loss of health have been produced by the attempt to remove old abscesses or other chronic diseases of this organ, and there are few of its lesions in which it is still not thought advisable to try small or full doses of the drug.

"On this subject it is unnecessary to dwell at present. The real question is, whether the evidence is satisfactory, or whether further researches are necessary. On this and many other topics connected with therapeutics, what we require are not unfounded assumptions and vague speculations, but positive knowledge, based on unquestionable data. These we have furnished, and consider them amply sufficient to demonstrate the fallacy of the opinion everywhere prevalent as to the cholagogue action of mercury."*

In discussing the subject we shall assume with the committee, that the action of mercury in man and dog is probably the same, but we deem such experiments only calculated to establish the *physiological* effects of the agent. We repeat, that the only definite conclusion to which they could logically lead is, that mercury, when administered to persons *in health* diminishes the secretion of bile. We take exception to the application of this truth, experimentally demonstrated, *in the denial of its therapeutical effects and uses in hepatic derangements.* If they had been content with the establishment of the *physiological effects* of the medicine, their labors would have been regarded by an appreciative profession as a monument of the zeal and industry of the experimenters; but when they were made to supersede the convictions firmly established by long-continued and enlightened clinical observation of the use of mercury in hepatic disorders, every individual, himself an experimenter, applies the test of his own therapeutical observation. Let it not be said that the committee simply state "that the notion of its doing good by *increasing* the biliary secretion is untenable." They make a sweeping denial of the "cholagogue action of mercury," which, of course, embodies its general employment in hepatic disorders. Regarding the evidence as unsatisfactory and insufficient to lead to this important practical end, we take

*See American Journal of Medical Science — vol. lviii, July No., 1869, pp. 234, 235, 236.

issue with the committee, basing our objections on the following propositions:

PROPOSITION I.—*Clinical Observation, rather than Vivisections, affords the most certain data from which the Therapeutical Effects and Uses of Remedies are to be drawn.*

PROPOSITION II.—*The Physiological Effects of Medicines do not always indicate what their probable Therapeutical Effects will be: The exceptions are so numerous as to enforce the greatest caution how we draw conclusions from data physiologically obtained, particularly when the latter would contravene well-established uses of remedies.*

PROPOSITION III.—*That the Committee fly the issue which they have precipitated when, in reply to this, they assume to require "Objectors" to "prove that, in any case whatever, mercury can increase the biliary secretion in man."*

PROPOSITION IV.—*That the Hepatic Uses of Mercury are not dependent upon a simple increase of the bile. It is not so regarded by those who employ it.*

PROPOSITION V.—*How Observations on the Effects of Mercury upon the liver may be made.*

---

PROPOSITION I.—*Clinical Observation, rather than Vivisections, affords the most certain data from which the Therapeutical Effects and Uses of Remedies are to be drawn.*

The overshadowing importance that the committee attach to the conclusions arrived at by the mode of experiment adopted, renders it necessary that some definite estimate of the value of vivisections in their real relations to, and bearings upon, practical medicine should, if possible, be made. The influence that they assign to them, amounts to a dogmatic control over the approaches of therapeutic measures, otherwise deemed appropriate, because it nullifies in advance, *all possibility* of different results in pathological conditions. Professor Stille says, that "in therapeutical questions, the convictions of competent judges must be allowed, to some extent at least, to acquire the authority which belongs to demonstration in the exact sciences." Admitting the competency of the profession, particularly those who reside in malarial sections, where hepatic disorders are most frequently seen, to decide this therapeutical question on the basis of enlightened observation in the use of mercury, it may well be feared that an inlet to error in medicine is opened, by not assigning true limits to physiological investigations of this character. The latter can be, at best, only *suggestive* in character—affording results to be taken on trial by clinical observers, precisely as when some newly discovered agent, or new application of an old remedy, is announced. It is tried in the crucible of therapeutic experimentation, which is clinical medicine being exemplified, where its asserted virtues are to be confirmed, rejected, or developed. It happens in the case of this medicine, that clinical observation has forestalled the suggestions of the experimental theorist, by establishing almost universally, strong convictions of the wholesome, corrective influence of mercury upon hepatic derangements. We hold, therefore, with Dr. Alison, "that a disagreement between a newly-broached pathology, and the practical experience of all time, is a much better reason for setting aside the new pathology than the old practice."—*Essentials of Practical Medicine, page* 22.

Medicine, we recognize as an experimental science, but the experiments are no less *therapeutical* than physiological and pathological. Mutually beneficial and reactive, no one of them can be exalted to the higher position of dictating authoritatively to the others. Each, in its own sphere, may be regarded as absolute, but relatively impotent, when not in harmony with its respective neighboring departments. There are laws in each of them, better understood, and, perhaps, more easily conformed to, in physiology and pathology than in therapeutics (the latter, however, are none the less binding), which, when harmonized or brought into proper relations, secure for practical medicine its present, and an increasing degree of certainty. In this sense, we have on another occasion, defined "*Rational Medicine*," as "the *co-aptation of all physiological, pathological, and therapeutical laws*." The unbroken harmony of these several departments will constitute the glory of its perfection. That auspicious period, when therapeutics is to be found in perfect accord with physiology and pathology, doubtless the committee would gladly inaugurate, but we must remind them that *errors in the interpretation and application of physiological results to the therapeutical department*, are as likely to retard as simple errors of observation in practice. If they would but believe it, the universal conviction of the utility of mercury in hepatic derangements, and the implied influence of the agent upon the organ itself, comes with all the force of a demonstration, the more so, indeed, since they declare it *impossible* that, in the present state of physiological chemistry, "direct observations" as to the influence of mercury upon the liver in man can be made. This being so, let us rightfully accord to clinical medicine the position of an arbiter, expecting that the judgment rendered will be based upon more certain data as to the therapeutical effects and uses of remedies than can be obtained elsewhere. The enlightened experience of the profession is both a test and the expression of this fact, perhaps, affording, also, a sure means of stamping upon similar experimental efforts the really limited value that they possess. Good, in a physiological sense, in their therapeutical bearings, they are null and void, as against clinical observations. Now then, which shall we accept—the judgment of the profession, clinically sustained, or the conclusion of the committee, experimentally derived, namely, that since mercury diminishes the secretion of the bile in man and dog, *in health, therefore, it will do the same in disease?* This brings us to the discussion of

PROPOSITION II.—*The Physiological Effects of Medicines do not always indicate what their probable Therapeutical Effects will be: The exceptions are so numerous as to enforce the greatest caution how we draw conclusions from data physiologically obtained, particularly when the latter would contravene well-established uses of remedies.*

We understand by the *physiological* effects of medicines such as are produced upon healthy persons, or irrespective of disease—the *therapeutical* effect is the curative result contemplated, or effected by its administration. Each has its relative and distinct value. The former are important, as being not only suggestive of probable therapeutical uses, (although the result expected is not always obtained,) but they constitute the best basis for the classification of medicines, all articles having similar tendencies to, or affinities for, particular organs or parts of the system, being brought together in the same class.

In the case of new remedies, a knowledge of their physiological properties is indispensable; it is an essential prerequisite to safe experimentation, or trial, in disease in the human. Much success has been obtained in this field of investigation. The materia medica has been greatly enriched in recent times in this way, by the addition of such articles as chloral, bromides, sulphites, etc., whose therapeutical properties known, and widely appreciated, are the valued results of scientific inqury. They are forcible illustrations of the great value of chemistry and physiology, with the aid of direct experiments, to practical medicine, and point unmistakably to the great truth, sooner or later to be realized by all practitioners, that empirical observation, with its results accidentally obtained, has given place to scientific inquiry, as the means of adding to the resources of the therapeutist.

With old remedies, which are emphatically the gift of experience, by studying their physiological effects, where the therapeutical are already known, light is thrown upon the mode of action, and explanation afforded for particular effects that would otherwise remain obscure.

We can illustrate it in this way: Veratrum viride is known to reduce the frequency and force of the pulse; empirical observation disclosed the fact, its properties as an arterial sedative, being universally recognized. This effect and its application to the treatment of disease form, however, the limit of empirical observation. Science takes up the subject at this limit, and asks *how* the heart's action is thus controlled. The sphymograph first confirming the above observation, the experimenter,* Horatio C. Wood, jr., by a section of the pneumogastric nerves demonstrates that it was by reason of the effect of the alkaloid (viridia) on the *sympathetic* nerve in its relations to this organ: thus supplementing clinical experience as to the power or property of the substance to control the heart, with a certain knowledge of its mode of action.

*See Physiological Action of Viridia, etc., by H. C. Wood, jr.—American Journal of Medical Sciences, January No., 1870, vol. lix.

So, also, digitalis, one of the oldest and best known remedies, if not by direct experiment, at least by close scientific study, has acquired new value in its present application as a "*cardiac-tonic*," in a stage or state of organic affections of the heart, almost, if not quite, out of reach of other measures: its power to promote the physiological irritability of the organ, through an influence upon the sympathetic ganglia, is the key to its application and efficacy under such circumstances.

Many other instances might be cited, but these are sufficient to indicate the sense of obligation felt by the therapeutist to his scientific allies. Enough, also, to show that we are not thoughtlessly nor ignorantly seeking to depreciate such efforts at the elucidation of occult subjects in therapeutics. Unfortunately, however, in the case of many valuable remedies, their *known* therapeutical properties are in no wise reflected or indicated by their *apparent* physiological effects. The latter are an absolute nullity, so far as anything arising therefrom might suggest their employment in morbid conditions, over which experience shows them to possess almost certain control. This fact, which compels admission by all, is sufficient to show the impotency of the experimentalist to decide upon the efficacy of remedial agents in pathological conditions, and should caution the profession to beware how they yield important practical convictions to his decision.

Certain classes of medicine are peculiarly obnoxious to this observation, embracing articles of daily use, many of them, indeed, the most potential and specific found in the materia medica. There is so little in the history of their physiological effects to prompt useful employment in disease, that, without their known efficiency, acquired by actual observation, they would be literally useless. Of these, alteratives and tonics may be selected as examples.

By way of illustrating our proposition, we would inquire, How shall we test the value of tonics, except upon sick persons, where tone or strength is deficient? Have tonics any *physiological* effects, properly so called? Any distinctive, prominent, dynamic property, likely to lead to, and become suggestive of, important therapeutical uses? We trow not.

Iron is a restorative tonic—clinical observation shows it indispensable in anæmia and impoverished states of the blood at large—but how could *experiments on healthy* animals indicate this therapeutic want of the blood, in the absence of physiological and pathological induction which demonstrate it the imperative want of the system in the above specified conditions? It is a specific, but its properties are beyond demonstration by such a process. Again: alcohol is a stimulant and tonic—it increases the force and *frequency* of the pulse in health; when employed therapeutically in weak subjects, it not only augments the force, but *reduces the frequency* of the pulse, under due support infused. Here there is a therapeutical end not surely indicated by an agent which, according to the recent investigations of Dr. Parkes, when given to a person in health, only "*labors*" the heart. In effect, *it is arterial sedation produced by an arterial stimulant*—a paradox, but true, nevertheless. In health, it has been styled the "genius of degeneration"; in disease, properly adapted, it is the harbinger of warmth, vigor, yea, even life.

What shall we say of cod liver oil in wasting diseases? Physiological considerations establish its value as aliment, in common with other oleaginous substances, but don't mistake this for its physiological effects as a medicine, likely to be developed by experiments upon healthy individuals. It has none.

Turning to alteratives next, the roll-call summons mercury, iodine, arsenic to the stand. In their physiological effects, disturbers, emphatically *destructives, poisons,* symbolizing impoverishment, degeneration, and waste; in their therapeutical effects and uses recognized as improvers of nutrition, blood-purifiers, specifics, each in its own sphere, therapeutically defined. What could be more opposite, antipodal? How did we obtain knowledge of the latter? Members of the committe, who would dispose of the well-nigh universal conviction of medical men as to the cholagogue influence of mercury, by physiological experiments on *nine* dogs, who bequeathed to science a knowledge of the properties and uses of these indispensable agents? Empiricism is the benefactor of the race in this instance. Paracelsus has been styled the "Prince of Quacks," we believe. If the profession is to be taunted with the remark, that the claims of mercury originate in a mere "*notion*," contained in some "*vague statement*" of this empiric, it may reconcile them to its longer indulgence (at least until shown to be untrue) to place beside it the further fact that empiricism has been the common foster-mother of all the sciences. Hippocrates, in lieu of the empirical practice "hereditary in his family," added reason to observation, thus laying the sure foundation of rational medicine, pervading which, when rightly understood, will be found a wholesome degree of eclecticism, which prompts it to take the good wherever it is found. Hence we are in debt to empirics, "old women," *et id omne genus*, as well as scientists.

But need we pursue the subject further? The facts and illustrations cited fully establish, we think, the proposition under discussion, and authorize the statement that *although the mercury may diminish the biliary secretion in healthy dogs and man, it does not, therefore, follow that the same effect will ensue in the diseases of man as at present apprehended.*

( *To be Continued* )

